# New protective battle-dress impregnated against mosquito vector bites

**DOI:** 10.1186/1756-3305-3-81

**Published:** 2010-09-01

**Authors:** Cédric Pennetier, Joseph Chabi, Thibaud Martin, Fabrice Chandre, Christophe Rogier, Jean-Marc Hougard, Frédéric Pages

**Affiliations:** 1 Institut de Recherche pour le Développement (IRD), UR016 CCPV/UMR MIVEGEC, and Centre de Recherche Entomologique de Cotonou (CREC), Cotonou, Bénin; 2 Centre de Coopération International en Recherche Agronomique pour le Développement (CIRAD), UR 103 HORTSYS, Montpellier, France; 3 Institut de Recherche Biomédicale des Armées, UMR 6236, Marseille, France

## Abstract

**Background:**

Mixing repellent and organophosphate (OP) insecticides to better control pyrethroid resistant mosquito vectors is a promising strategy developed for bed net impregnation. Here, we investigated the opportunity to adapt this strategy to personal protection in the form of impregnated clothes.

**Methods:**

We compared standard permethrin impregnated uniforms with uniforms manually impregnated with the repellent KBR3023 alone and in combination with an organophosphate, Pirimiphos-Methyl (PM). Tests were carried out with *Aedes aegypti*, the dengue fever vector, at dusk in experimental huts.

**Results:**

Results showed that the personal protection provided by repellent KBR3023-impregnated uniforms is equal to permethrin treated uniforms and that KBR3023/PM-impregnated uniforms are more protective.

**Conclusion:**

The use of repellents alone or combined with OP on clothes could be promising for personal protection of military troops and travellers if residual activity of the repellents is extended and safety is verified.

## Background

Mosquito-borne diseases are a permanent threat for both travellers and troops deployed in tropical areas [1]. Soldiers or tourists returning from regions of dengue or chikungunya transmission run the risk of importing the disease to their homeland potentially causing new outbreaks [2-5]. Personal protection is currently the only way to protect people against vector-borne diseases transmitted by diurnal mosquitoes such as dengue fever and chikungunya. The available methods are: topical use of repellents, pyrethroid treated clothes, insecticide vaporizers and mosquito coils [6]. These methods act by preventing the contact between humans and mosquito vectors, with the exception of the insecticide vaporizers which also aim to kill mosquitoes entering dwellings. The repellent and irritant properties of insecticides are therefore essential in these personal protection strategies [7,8]. Indeed, the pyrethroid insecticides are preferentially used in these strategies due to their irritant (for the majority of them) [7] and repellent (for the most volatile ones) properties [8].

Protective clothes are usually impregnated with permethrin which is one of the most repellent pyrethroids. Commercial products are available to travellers in the form of permethrin-based sprays for clothes. Other major users of permethrin-impregnated clothing are military troops deployed to tropical areas endemic for vector borne diseases. Personal protection is the only means to prevent contact with both diurnal and nocturnal disease vectors when sleeping under bed nets is not always possible. For these reasons, the French army developed long-lasting permethrin-treated battlefield uniforms (BFUs) [9]. Many studies have demonstrated the effectiveness of permethrin-impregnated BFUs in combination with skin repellents [10-15]. The World Health Organization (WHO) also recommends the impregnation of clothes with repellents [6]. From World War II to the 1980's, military fatigues were impregnated with repellents to protect soldiers against scrub typhus and mosquito borne diseases. Early products (like dimethyl-phthalate) offered a very short protection time and, until the eighties, DEET was the recommended product for fabric impregnation [16-18]. After this period DEET was replaced by permethrin that better resists washing and lasts longer on fabrics [19-22]. Slow-release repellent formulations are currently under development; however none are so far available [23].

Resistance mechanisms to pyrethroids (metabolic and target-site mutations, i.e. *Kdr *mutations) are now widespread among mosquito vectors. It has already been shown that irritation of *Kdr *resistant mosquitoes is reduced by pyrethroid insecticides [24]. The authors demonstrated that this decrease in irritant sensitivity elicits resistant mosquitoes to stay longer on pyrethroid impregnated bed nets, increasing the insecticide dose they get by tarsal contact until a lethal dose is obtained. For BFUs the mosquito does not actually have to touch the material to reach the uncovered skin parts such as the hands. In this case, the protective effect of treated BFUs is based on the irritant property of permethrin that limits the contact between the mosquito and human (even for exposed skin). Thus, what would be the impact of such pyrethroid resistance mechanisms on the efficacy of permethrin-treated clothes? One report highlighted a loss of efficacy of permethrin-impregnated BFU's against *Kdr*-resistant *Anopheles gambiae*, the major malaria vector, in Côte d'Ivoire [25]. To overcome such limitations for personal protection, we propose to explore the opportunity to use an alternative strategy recently developed to maintain the efficacy of impregnated bed nets against pyrethroid resistant *An. gambiae *[26].

This new strategy consists of associating a synthetic repellent with a non-pyrethroid insecticide to recreate features of pyrethroids, most importantly irritancy to pyrethroid-resistant mosquitoes [26-29]. Mixtures of repellent and organophosphate insecticide (OP) showed irritant properties, knock-down and mortality effects similar to deltamethrin against susceptible mosquitoes and higher than deltamethrin against *Kdr*-resistant mosquitoes [26,29]. In this study we adapted this new strategy for personal protection using impregnated clothes. The aim of the study was to compare the efficacy of fatigues impregnated with the repellent KBR3023 alone or combined with the organophosphate, pirimiphos-methyl (PM) *versus *the standard permethrin-treated uniforms (unwashed and washed 10 and 20 times) against *Aedes aegypti*, the main vector of dengue.

## Materials and methods

### Vector Collectors

The study was conducted in Ketonou (6°42N; 2°54E), a village situated North-East on the Nokoué lake, 30 Kms from Cotonou in experimental huts [30]. Six male inhabitants of Ketonou, previously hired by the Centre de Recherche Entomologique de Cotonou (Cotonou, Benin) for participation in vector studies and vaccinated against yellow fever, were enrolled into the current study after giving informed consent to test the impregnated BFUs.

### Battle Field Uniforms (BFUs)

The BFUs provided by the French Army, were designed for tropical areas (composition 65% cotton and 35% polyester). ATHANOR S.A. (Vieux- Thann, France), a French company, was in charge of the process of permethrin impregnation. Standard BFUs were industrially treated with permethrin 25/75 (25% *cis*-isomer and 75% *trans*-isomer) 1250 mg/m^2^. We manually impregnated one uniform with the repellent KBR3023 (10 g/m^2^) alone and one with KBR3023 (10 g/m^2^) in combination with PM (pirimiphos-methyl 150 mg/m^2^) (KBR3023/PM). We purchased the PM emulsifiable concentrate (EC) named 'Pirigrain 250' from the company CGI (Compagnie Générale des Insecticides, France), containing 25% PM. The company Osler, France, kindly gave us the KBR3023 formulation containing 25% KBR3023. An identification number was allocated to each jacket and trouser. Each volunteers used every uniform following a Latin square. Two batches of BFUs (permethrin-treated and untreated) were grouped into three loads for washing, with one load washed 10 times (X10 in the Results tables), another 20 times (X20 in the Results tables) with the last load not washed. Washing and drying was done according to an International Organization for Standardization (IOS) procedure (NF EN ISO 6330): 60°C in a washing machine with detergent for colour fabrics but without perborate, followed by drying in a clothes dryer. Washings of permethrin-treated BFUs were carried out under the supervision of the Service Central d'Etude et de Recherche du Commissariat de l'Armée de Terre (SCERCAT) to respect IOS standard n°105. The KBR3023 and KBR3023/PM BFUs were not subjected to washing.

### Study Design

The vector collectors wore long-sleeved uniforms and a pair of athletic shoes laced up with socks covering the feet and ankles. Each stayed individually in one of the six huts at dusk and collected mosquitoes 2 hours later. For each mosquito collection session, a treatment (Status of BFUs: impregnated, non-impregnated, washed, non washed, etc.) was allocated to each collector and to his experimental hut. Seven collecting sessions (9^th^, 11^th^, 12^th^, 14^th^, 15^th^, 16^th ^and 19^th ^February 2007) were conducted allowing each volunteer to wear each type of BFU at least once following a blind study design and strict timetable. We used the *Ae. aegypti *SBE strain originating from Benin which is free of any detectable resistance mechanisms [31]. During each session, batches of SBE strain (60 *Ae. aegypti *females per batch) were released at dusk in each experimental hut (5.30 p.m). Mosquitoes were collected (by aspirators) two hours later at 7.30 p.m. from each hut. Female mosquitoes were classified according to feeding status, whether dead or alive and whether they have been caught in the veranda or the room. Live mosquitoes were kept for 24 h in plastic cups under standard insectary conditions, provided with honey-soaked cotton wool, before re-classifying as dead or alive.

### Statistical Analysis

The associations between the treatment and the mosquito status (dead/alive, blood-fed/unfed, and caught in the veranda/room) were tested using a Poisson regression model. We used a generalized estimating equation (GEE) approach for statistical analysis of clustered measures, *i.e*. measures made during the same night [32]. The GEE approach allows some departure from the hypothesis about the distribution of the dependent variable and gives robust estimates of the regression coefficient, taking into account the interdependence of mosquitoes caught on the same night. The numbers of mosquitoes caught with a particular status (dead/alive, feeding status or veranda/room caught) were treated as dependent variables and the overall number of mosquitoes caught in each case (*i.e*. room plus veranda) as the exposure variable. Then, the exponentials of the regression coefficients are estimates of risk ratios for the mosquitoes to be caught with the considered status. Data were analysed using STATA 9.0^® ^statistical package (StataCorp 2005) software program.

### Ethical Considerations

All volunteers were recruited after obtaining informed written consent. A medical doctor was on hand during the trial to respond to any side effects of the BFUs. The protocol received approval from the ethics committees of The Ministry of Health, Benin, and Institut de Recherche pour le Développement, France.

## Results

During the seven collecting sessions, around 2520 *Ae. aegypti *females were released with 2023 females re-captured (~80%). The results are presented in three tables illustrating the effect of impregnated BFUs on exophily rate, blood feeding inhibition rate and mortality rate, respectively.

### Induced exophily (Table 1)

Permethrin-treated BFUs, unwashed and washed 20 times significantly increased the exophily rate (p < 0.05) while permethrin-treated BFUs washed 10 times did not (p > 0.05; relative to the untreated BFU). The increase in exophily was significant for the BFUs treated with both KBR3023 alone (p < 0.001) and in combination with PM (p < 0.001).

**Table 1:** Exophily induced by BFUs on released *Ae. aegypti*.

	Untreated BFU	Unwashed permethrin-treated BFU	Permethrin-treated BFU washed 10 times	Permethrin-treated BFU washed 20 times	Unwashed KBR-treated BFU	Unwashed PM/KBR-treated BFU
**Total females caught**	351	300	260	317	391	404
**Exophily (%)**	28.39	36.76	30.12	37.15	43.63	43.51
**95% Confidence limits**	23.35-34.53	30.52-44.28	24.16-37.57	31.03-44.48	37.57-50,67	37.55-50.42
**Induced Exophily (%)**	-	11.43	1.57	11.77	20.6	20.73
**Relative Risk (RR)**	1	1.29	1.06	1.30	1.50	1.50
**95% Confidence limits**	-	1.04-1.60	0.80-1.30	1.06-1.60	1.30-1.90	1.30-1.90
**p**	-	p < 0.05	NS	p < 0.05	p < 0.0001	p < 0.0001

The relative risk (RR) represents the increase factor to be caught in the verandah trap due to the treatment on BFUs.

### Blood feeding inhibition (Table 2)

**Table 2:** Blood feeding inhibition induced by BFUs on released *Ae. aegypti*.

	Untreated BFU	Unwashed permethrin-treated BFU	Permethrin-treated BFU washed 10 times	Permethrin-treated BFU washed 20 times	Unwashed KBR-treated BFU	Unwashed PM/KBR-treated BFU
**Total females caught**	351	300	260	317	391	404
**Total females blood fed**	223	84	90	126	92	61
**Blood fed females (%)**	63.53	28.00	34.61	39.74	23.52	15.09
**95% Confidence limits**	55.50-72.15	22.74-34.83	27.94-42.27	33.36-47.28	19.10-28.76	11.74-19.38
**Blood Feeding Inhibition (%)**	-	56.25	45.31	37.50	62.50	76.56
**Relative Risk (RR)**	1	0.44	0.54	0.62	0.37	0.23
**95% Confidence limits**	-	0.35-0.55	0.43-0.67	0.51-0.75	0.29-0.45	0.18-0.30
**p**	-	p < 0.0001	p < 0.0001	p < 0.0001	p < 0.0001	p < 0.0001

The relative risk (RR) represents the decrease factor of blood feeding rate due to the treatment on BFUs.

On average 64% (56.5-68.6) of recaptured females took a blood meal on the collectors wearing untreated BFUs (controls). Only 28% (22.74-34.83) of the females took a blood meal when the collectors wore unwashed permethrin-treated BFUs, indicating that the initial permethrin concentration inhibited 56.25% of blood meals (BFI, Table 2). The blood feeding inhibition (BFI, Table 2) decreased significantly with washing to 37.5% for the permethrin-treated BFU washed 20 times. KBR3023-treated BFU inhibited as many bites (62.50%) as unwashed permethrin-treated BFU (56.25%, Confidence Intervals overlap) while the KBR3023/PM-treated BFU prevented significantly more bites (76.56%). There was no significant difference between KBR3023 used alone or combined with PM (Confidence Intervals overlap).

### Mortality (Table 3)

The unwashed permethrin-treated BFU induced significantly more mortality (11.4%) than the permethrin-treated washed 10 times and the KBR3023/PM-treated BFUs (3.6% and 2.2% respectively). There was no significant difference between the permethrin-treated washed 10 times and the PM/KBR-treated BFUs (Confidence Intervals overlap). The others treatments did not induce any significant mortality relative to the control.

**Table 3:** Mortality induced by BFUs on released *Ae. aegypti*.

	Untreated BFU	Unwashed permethrin-treated BFU	Permethrin-treated BFU washed 10 times	Permethrin-treated BFU washed 20 times	Unwashed KBR-treated BFU	Unwashed PM/KBR-treated BFU
**Total females caught**	351	300	260	317	391	404
**Total dead females**	1	35	10	5	8	10
**Mortality (%)**	0.28	11.66	3.84	1.58	2.04	2.48
**95% Confidence limits**	0.04-2.02	8.43-16.30	2.06-7.14	0.66-3.80	1.02-4.09	1.33-4.60
**Corrected mortality (%)**	-	11.4	3.6	1.3	1.8	2.2
**Relative Risk (RR)**	1	40,03	13.16	5.43	7	8.49
**95% Confidence limits**	-	5.50-288.90	1.69-102.70	0.63-46.79	0.87-56.08	1.09-66.27
**p**	-	p < 0.0001	p < 0.05	NS	NS	p < 0.05

The Relative risk (RR) that represents the increase factor of mortality rate due to the treatment on BFUs.

## Discussion

In using a chemical on clothing, we aim to repel or irritate the mosquitoes that try to land and probe even though there is a physical barrier provided by the fabric. Our results showed that using an insecticide is not essential to protect people wearing impregnated clothes. Indeed, KBR3023-impregnated BFUs protected the user as much as permethrin-treated BFUs (56.25% of bites inhibited, BFI in Table 2). The BFUs impregnated with the repellent KBR3023 did not induce more exophily than permethrin. Siegert *et al*. [33] showed that permethrin-impregnated bed nets reduced landing attempts and increased frequency of flight compared to deltamethrin-treated nets. They concluded that permethrin acts more as a disengaging agent than an insecticide. Indeed, this kinetic disengagement resulted in limited mortality [33,34]. Considering our entomological data, we can conclude that KBR-impregnated clothes acted in the same way as permethrin, with equal efficiency.

The new strategy using repellents in combination with PM conferred better protection than permethrin treated BFUs and slightly (but not significantly) better than the KBR used alone. The exophily induced by the mixture is no different from that induced by the KBR alone, probably because the PM is not a repellent or irritant. The mortality induced by the mixture is also no different to the KBR alone even though a strong synergistic interaction has already been demonstrated between the two compounds [27,28]. Indeed, strong synergistic interactions have been previously observed with bed nets impregnated with the same mixture in the same experimental conditions (experimental huts against *An. gambiae*). This suggests that, during the host approach, the mosquito contact with a bed net differs sufficiently from contact with the BFU triggering different behavioural consequences [27,28]. Nevertheless, in the present study, the blood feeding inhibition is slightly higher with the mixture, suggesting that PM could contribute to increase the irritant and/or disengaging effect of the treated BFU [29].

It is interesting to note that when collectors wore untreated BFUs, the blood feeding rate was 26% less than observed in previous studies in which the control blood feeding rates were as high as 90% for *Ae. aegytpi *[31] and *An. gambiae *[13]. This difference could be due to the study design. Indeed, in our protocol the collectors wore socks during the trial in contrast with the previous studies [13,31]. Body part preferences for different mosquito species have been well studied [35,36]. *Aedes *species are known to prefer ankle areas to feed on. This highlights that the physical barrier constituted by clothes could prevent a significant amount of mosquito bites. Nevertheless it could be interesting to repeat the study against mosquito vectors with different landing preferences. For example, De Jong & Knols [35] showed that *An. atroparvus *females prefer landing on the human head while *An. gambiae *prefer landing on the ankles. Personal control strategies must be adapted to the behavioural preferences of the mosquito populations.

As mentioned in previous studies [28,29], the first limitation of this new personal protection opportunity is the short residual effect of repellents. Companies are, however, currently developing new formulations or impregnation processes to overcome this difficulty. Encouraging results have already been observed with the standard repellent DEET which lasted several months on bed nets [23]. These promising results should lead companies to develop long-lasting technologies with repellents and more importantly mixtures due to the ongoing threat of pyrethroid resistance [37]. The efficacy of KBR3023-impregnated clothes (alone and in mixtures) now must be tested against pyrethroid resistant mosquitoes.

Finally, it is obviously safer to impregnate clothes with repellents than with permethrin. However as any mixture of chemicals has to be considered as a new compound, it is essential to investigate the safety for the user of clothes impregnated with OP-repellent mixtures. In the literature, studies already showed the potential toxicity of topical application of DEET with permethrin-treated clothes. DEET could potentially increase the skin penetration rate of permethrin [38-40]. But as far as we know, no data concerning the toxicity of clothes impregnated with both repellents and OP is available.

To conclude, our results highlight once again the potential for using combined repellents and non-pyrethroid insecticides to prevent vector-human contact. Their potential efficacy against pyrethroid resistant mosquitoes has already been demonstrated in terms of irritancy, repellence, blood feeding inhibition and mortality in the form of treated bed nets [26-29]; however bed nets and clothes probably disturb the human host approach differently.

This study confirms that impregnated clothes are effective in protecting against diurnal vector bites and can be recommended to protect people during disease outbreak scenarios or travellers and soldiers deployed to endemic areas. Their use by travellers can reduce their risk of being infected by dengue and chikungunya viruses and therefore the risk of disease introduction into the now susceptible areas of southern Europe. To limit the exposure of travellers to pesticides and to provide consistent protection, industrially impregnated clothes would be preferred to personal application direct to the skin. There is now a need to assess the efficacy and tolerability of the commercially available impregnated clothes alongside novel OP-repellent mixtures for the civilian population [41-44].

## Competing interests

The authors declare that they have no competing interests.

## Authors' contributions

CP, JMH, CR and FP designed the study. JC and CP carried out the experiments. CR and FP analyzed the data. CP and JC drafted the manuscript. TM, FC, CR, JMH and FP critically revised the manuscript. All authors read and approved the final manuscript.
